# Graft dysfunction is associated with late CMV infection after kidney transplantation

**DOI:** 10.3389/frtra.2025.1647725

**Published:** 2025-11-14

**Authors:** Angela Zeng, Katherine Barraclough, Michael Lian, Rosemary Masterson, Peter Hughes, Kevin V. Chow

**Affiliations:** 1 Department of Nephrology, Royal Melbourne Hospital, Melbourne, VIC, Australia; 2 Department of Medicine (RMH), Faculty of Medicine, Dentistry and Health Sciences, The University of Melbourne, Melbourne, VIC, Australia

**Keywords:** late CMV infection, renal failure, kidney transplantation, cytomegalovirus infection, graft survival

## Abstract

**Background:**

Cytomegalovirus (CMV) causes significant morbidity and mortality following kidney transplantation. Late CMV infection (≥2 years post-transplant) is uncommon, and its risk factors and outcomes may differ from earlier infection.

**Methods:**

We conducted a single-centre retrospective study of kidney transplant recipients between 2009 and 2019. Patients were grouped by CMV status: no infection, early infection (<2 years post-transplant), and late infection (≥2 years post-transplant). Clinical characteristics and outcomes were compared.

**Results:**

Donor-positive/recipient-negative (D+/R−) serostatus was observed in 105/710 (14.8%) patients without CMV, 28/42 (66.7%) with early CMV, and 2/28 (7.1%) with late CMV (*p* < 0.001). Prior rejection occurred in 5.9%, 16.7%, and 10.7% respectively (*p* = 0.017). Median serum creatinine was 113.0, 127.5, and 219.5 µmol/L respectively (*p* < 0.001). CMV serostatus was significantly associated with early infection (*p* < 0.001), while only serum creatinine was associated with late infection (*p* = 0.003). Trends were seen toward better one-year patient survival (97.6% vs. 85.7%, *p* = 0.051) and graft survival (88.1% vs. 71.4%, *p* = 0.073) after early vs. late infection.

**Conclusions:**

Risk factors for CMV infection differ by timing post-transplant. Renal dysfunction may be a key predictor of late infection. identifying at-risk patients may support targeted surveillance and improve long-term outcomes.

## 1 Introduction

Cytomegalovirus (CMV) is an important opportunistic pathogen that causes significant morbidity and mortality following kidney transplantation (1, 2). CMV seronegative recipients of organs from CMV seropositive donors (D+/R−) are at the greatest risk of infection, with most historical cases, prior to the use of prophylaxis, occurring within the first three months post-transplantation (3). The advent of widespread use of CMV prophylaxis in these high-risk patients has resulted in a shift toward most infections occurring at later time points, after the cessation of prophylaxis (4). The term “late infection” has not been well defined and has been used in an arbitrary fashion in the past. It has often been used to refer to cases of CMV infection which occur after the completion of prophylaxis, however the vast majority of cases still occur within the first 12 months post-transplantation (5, 6). Late CMV infection occurring beyond the first-year post-transplantation is much less common and has only been reported sporadically in the literature (7–9).

Unlike with early CMV infection, the lack of clearly defined risk factors for the development of late infection results in an inability to effectively identify at risk patients who might benefit from closer observation and/or reduction in immunosuppression regimens to prevent infection. The limited number of case reports and case series examining late CMV infections in solid organ transplant recipients suggest that the risk factors associated with very late CMV infection might be quite different to those of the traditional risk factors associated with earlier infection. However, these remain poorly understood as previous studies have been limited by small numbers of cases and heterogeneous methodologies. Additionally, the outcomes following late CMV infection remain poorly understood.

In this study we report patient characteristics and outcomes in patients with late CMV infection after kidney transplantation compared to those with early post-transplant infection. We have defined late CMV infection as those which occur ≥2 years post-transplant. This definition has been selected to ensure clear separation between genuinely late infections and earlier post-prophylaxis episodes and is consistent with previous literature (10, 11).

## 2 Materials and methods

### 2.1 Study design

We conducted a single-center retrospective cohort study at the Department of Nephrology, Royal Melbourne Hospital, Victoria, Australia. The study included adult kidney transplant recipients (aged ≥18 years) who received care between January 2009 and September 2019. Data were obtained from the departmental database and institutional electronic medical records.

### 2.2 Ethics approval

This study was approved by the Royal Melbourne Hospital Human Research Ethics Committee (QA2019136).

### 2.3 Study population

All adult kidney transplant recipients who received care at the Royal Melbourne Hospital during the study period were eligible for inclusion. Patients were classified according to CMV infection status and timing of infection post-transplant. A comparison cohort of transplant recipients without CMV infection during the same period was also included. Data collected included age, sex, previous kidney transplants (graft number), graft type, CMV serostatus, serum creatinine and history of rejection.

### 2.4 CMV prophylaxis protocol

During the study period, CMV prophylaxis for transplant recipients at our institution consisted of valganciclovir 900 mg daily (adjusted for renal function) for 6 months in CMV-seronegative recipients (R−) of CMV-seropositive organs (D+), and for 3 months in CMV-seropositive recipients (R+). Prior to May 2014, CMV-seronegative recipients (R−) of CMV-seronegative organs (D−) received no prophylaxis; thereafter, they received valganciclovir 900 mg daily (adjusted for renal function) for 1 month.

### 2.5 Definition and classification of CMV infection

CMV infection was defined by a positive CMV polymerase chain reaction (PCR) ≥1,000 IU/ml (equivalent to 3 log_10_ IU/ml) from a peripheral blood sample. Viral loads below 2–3 log_10_ IU/ml are generally considered unlikely to lead to CMV disease when used as a threshold for pre-emptive therapy (12). Additionally, low-level viraemia may be due to “blips”; however signals above 910 IU/ml have been associated with an increased risk of subsequent genuine CMV infection, supporting the use of our selected threshold (13).

Patients with CMV infection were further stratified by the time of onset post-transplant: early CMV infection was defined as <2 years post-transplant, and late CMV infection as ≥2 years post-transplant. This time frame was selected to avoid including cases of post-prophylaxis, delayed onset disease, which has been shown to occur up to 2 years post-transplant following 6 months of prophylaxis in D+/R− patients (14). Prior research has also suggested that CMV DNAemia occurring ≥2 years post-transplant may reflect a distinct clinical entity with specific risk factors (10).

### 2.6 Assessment of kidney function

Kidney function was assessed using serum creatinine. For patients with CMV infection, the serum creatinine value measured at the time of the first positive CMV PCR was used. For patients without CMV infection, the most recent available serum creatinine was used.

### 2.7 Statistical analysis

Baseline characteristics were reported as counts and percentages for categorical variables, and as means with standard deviation (SD) or medians with interquartile range (IQR) for continuous variables. Differences between groups were assessed using the chi-square test for categorical data and one-way ANOVA for continuous data.

Multivariate logistic regression was used to assess associations between variables and early or late CMV infection. Outcome measures, including patient and graft survival, were compared on a time-to-event basis using the log-rank test. Statistical analyses were conducted using GraphPad Prism software, with two-sided *P*-values <0.05 considered statistically significant.

## 3 Results

### 3.1 Patient population and characteristics

A total of 780 patients met the inclusion criteria for this study. These included 710 who had no evidence of CMV infection, 42 who had early CMV infection <2 years post-transplant and 28 who had late CMV infection ≥2 years post-transplant (Table 1). There were no significant differences in age at transplantation between the three groups (50.7 vs. 51.4 vs. 46.9 years, *p* = 0.273). The gender distribution between the three cohorts was also similar, with male recipients comprising 441/710 (62.1%) in the group without infection, and 23/42 (54.8%) in the early CMV group, and 16/28 (57.1%) in the late CMV group (*p* = 0.565).

**Table 1:** Comparison of the clinical characteristics between the patients with or without CMV infection; univariate analysis.

Clinical characteristic	No CMV	CMV infection^a^		*P* *
		Early (<2 years)	Late (>2 years)	
	(*N* = 710)	(*N* = 42)	(*N* = 28)	
Demographics				
Male	441 (62.1)	23 (54.8)	16 (57.1)	0.565
Age at Tx, years	50.7 (40.1, 60.3)	51.4 (39.9, 61.8)	46.9 (36.2, 57.6)	0.273
Age at CMV infection, years		52 (40.3, 62.6)	57.2 (43.1, 68.2)	0.194
Graft number				
1	587 (82.7)	34 (81.0)	26 (92.9)	0.350
≥2	123 (17.3)	8 (19.0)	2 (7.1)	
Transplant type				
Deceased Donor	446 (62.9)	31 (73.8)	15 (53.6)	0.407
Donation after Brain Death (DBD)	322 (45.4)	24 (57.1)	12 (42.9)	
Donation after Circulatory Death (DCD)	124 (17.5)	7 (16.7)	3 (10.7)	
Living donor	264 (37.2)	11 (26.2)	13 (46.4)	
Serostatus				
D+/R-	105 (14.7)	28 (66.7)	2 (7.1)	<0.001
Others	595 (83.8)	14 (33.3)	23 (82.1)	
Unknown	10 (1.4)	0 (0)	3 (10.7)	
Transplant characteristics				
Follow up time, days	1,929	1,610	4,605	<0.001
Time to CMV infection post-transplant, days ^b^		219 (131, 276)	3,136 (2,111, 5,183)	<0.001
HLA Mismatch ^c^ ^,^ ^d^	3.7 +/− 1.7	4.0 +/− 1.3	3.8 +/− 1.4	0.395
Rejection	42 (5.9)	7 (16.7)	3 (10.7)	0.017
Lymphocyte count (10^9^/L)^e^	1.7 (1.2, 2.3)	1 (0.7, 1.8)	0.6 (0.4, 1.2)	0.001
Tacrolimus (µg/L)^f^	4.9 (3.9, 6.5)	6.5 (4.9, 7.9)	3.6 (2.7, 4.9)	<0.001
BK virus infection	144 (20.3)	13 (31.0)	6 (21.4)	0.529
Time to BK virus infection post-transplant (days)	90 (62, 185)	92 (58, 901)	195 (89, 1,954)	0.169
Serum creatinine (µmol/L)	113.0 (91, 144)	127.5 (98.5, 187.5)	219.5 (148.8, 249.5)	<0.001

Values are represented as number (%) unless otherwise stated.

a Defined as blood CMV PCR ≥1,000 IU/ml.

b Median (IQR).

c Mean ± SD.

d HLA data missing in 3/710 CMV negative, 0/42 early CMV, 5/28 late CMV.

e Lymphocyte count data missing in 56/710 CMV negative, 20/42 early CMV, 13/28 late CMV.

f Tacrolimus data missing in 174/710 CMV negative, 4/42 early CMV, 11/28 late CMV.

* Based on one-way ANOVA, chi-square test, Log-rank (Mantel-cox) test, two tailed student's *t*-test.

Most patients in all three groups were first-time transplant recipients [587/710 (82.7%) vs. 34/42 (81.0%) vs. 26/28 (92.9%), *p* = 0.350], and most received kidneys from deceased donors [446/710 (62.9%) vs. 31/42 (73.8%) vs. 15/28 (53.6%), *p* = 0.407]. The degree of HLA A, B and DRB mismatch (out of 6) was comparable across groups (mean 3.7 vs. 4.0 vs. 3.8, *p* = 0.395).

The median time to onset of CMV infection was 219 days post-transplant in the early infection group compared with 3,136 days post-transplant in the late infection cohort (*p* < 0.001) (Supplementary Figure S1). The majority of early CMV infection was de-novo infection (R-) whereas this was true only in a minority of patients with late CMV infection (66.7% vs. 7.1%, *p* < 0.001) (Supplementary Table S1). 40/42 patients with early CMV and 26/28 patients with late CMV had CMV infection confirmed on multiple PCR tests, confirming the significance of their initial positive PCR (Supplementary Table 2). Of the remaining 4 patients, 3 were treated with valganciclovir or ganciclovir with resolution of viraemia by the time of the following test. The final patient (with late CMV infection) died from a malignant cause before a follow up test could be performed.

With respect to CMV serostatus, patients with the highest risk combination (D+/R−) comprised 28/42 (66.7%) of those with early CMV infection, but only 2/28 (7.1%) of those late CMV infection. In comparison, the D+/R− combination was found in 105/710 (14.8%) of patients with no history of CMV infection (*p* < 0.001). There were 42 subjects without CMV infection who had experienced at least one episode of allograft rejection (5.9%) compared with 7/42 (16.7%) and 3/28 (10.7%) in the early and late CMV infection cohorts respectively (*p* = 0.017). Rejection occurred at a median of 30 days before early CMV infection and 1980 days before late CMV infection.

Absolute lymphocyte count was higher in CMV negative patients compared with patients with early or late CMV infection (1.7 vs. 1 vs. 0.6 × 10^9^/L, *p* = 0.001). Trough tacrolimus levels were higher in subjects with early CMV infection compared to those with late CMV infection (6.5 vs. 3.6 µg/L, *p* < 0.001) reflecting our practice of reducing target tacrolimus levels as time elapses post-transplant). BK virus infection (another important opportunistic infection post-kidney transplantation) occurred at a similar rate in all three groups [144/710 (20.3%) vs. 13/42 (31.0%) vs. 6/28 (21.4%), *p* = 0.529], and at a similar time post-transplant (90 vs. 92 vs. 195 days, *p* = 0.169).

Kidney function at the time of CMV infection differed significantly between groups. The median serum creatinine was 127.5 µmol/L in the early CMV group and 219.5 µmol/L in the late CMV group, compared to 113 µmol/L in patients without CMV infection (*p* < 0.001). CMV “blips” (PCR <1,000 IU/ml) occurred in 14% of patients who did not ultimately develop clinical CMV infection (as defined by PCR ≥1,000 IU/ml). Whilst patients with early CMV infection had preceding blips at a similar rate, these occurred significantly more often in patients with late CMV infection [8/42 (19%) vs. 10/28 (35.7%), *p* < 0.001]. This may suggest that there may be a predilection for late CMV infection to have a more subacute and gradual onset compared to early CMV infection.

A multivariate logistic regression was performed to identify factors that were associated with early or late CMV infection (Table 2). Positive CMV serostatus was significantly associated with the development of early CMV infection (*p* < 0.001), while age at transplantation, sex, graft number, graft type and serum creatinine were not. There was a non-statistically significant trend toward an association with transplant type and early CMV infection. In contrast, serum creatinine was the only factor that was significantly associated with the development of late CMV infection (*p* = 0.003), whereas CMV serostatus was not (*p* = 0.190).

**Table 2:** Multivariate logistic regression of factors predictive of CMV infection.

Factor	CMV infection					
	Early			Late		
	OR^a^	95% CI^b^	*P*	OR	95% CI	*P*
Age	1	1.00–1.00	0.230	0.99	0.99–1.000	0.204
Gender (male)	1.45	0.71–2.98	0.306	1.67	0.72–3.85	0.222
Graft Number^c^	1.1	0.41–2.16	0.977	0.25	0.038–0.84	0.070
Transplant type (live donor)^d^	0.49	0.22–1.03	0.070	1.15	0.51–2.55	0.734
Recipient CMV Serostatus (non-D+/R−)	0.065	0.03–0.13	<0.001	2.81	0.75–18.9	0.190
Rejection history	2.61	0.74–8.52	0.120	0.18	0.02–1.24	0.110
Creatinine	0.998	0.995–1.00	0.200	1.004	1.001–1.006	0.003

a Odds ratio.

b 95% confidence interval.

c Graft number is the number of kidney transplants a patient has had (including the current one).

d Transplant type refers to live donor transplants or deceased donor transplants.

There was a weak but statistically significant positive correlation between serum creatinine and time post-transplant (Pearson correlation co-efficient R = 0.13, *p* < 0.001).

### 3.2 Patient outcomes after CMV infection

We examined outcomes of subjects with early and late CMV infection one year after CMV infection. There was a trend toward improved one-year patient survival in the early CMV infection group compared to the late infection group [41/42 (97.6%) vs. 24/28 (85.7%), *p* = 0.051], although this did not reach statistical significance (Table 3). A similar non- significant trend was observed for one-year graft survival [37/42 (88.1%) vs. 20/28 (71.4%), *p* = 0.073]. There was no difference in death-censored graft survival between groups [37/41 (90.2%) vs. 20/24 (83.3%), *p* = 0.454].

**Table 3:** Patient outcomes.

Outcome	No CMV	Early CMV	Late CMV	*P*
Outcomes one year after CMV infection				
Patient survival^a^		41/42 (97.6)	24/28 (85.7)	0.051
Graft survival^a^		37/42 (88.1)	20/28 (71.4)	0.073
Death censored graft survival^a^		37/41 (90.2)	20/24 (83.3)	0.454
Outcomes five years after transplantation				
Patient survival^a^	356/371 (96)	17/19 (89.5)	25/26 (96.2)	0.425
Graft survival^a^	345/379 (91)	15/22 (68.2)	24/26 (92.3)	<0.001
Death censored graft survival^a^	345/364 (94.8)	15/20 (75)	24/25 (96)	<0.001
Renal function at study completion				
Serum creatinine (µmol/L)	114 (92, 147)	123 (92, 175)	235 (126, 357)	<0.001
Time of creatinine post-transplant (days)	1,657 (783, 2,745)	942 (362, 2,126)	3,533 (2,440, 5,860)	<0.001

a Based on Log-rank (Mantel-Cox) test.

We next assessed outcomes of patients with no CMV infection, early CMV infection or late CMV infection at 5 years post-transplant. There were no significant differences in patient survival between the three groups [356/371 (96%) vs. 17/19 (89.5%) vs. 25/26 (96.2%), *p* = 0.425]. However, graft survival [345/379 (91%) vs. 15/22 (68.2%) vs. 24/26 (92.3%), *p* = 0.005] and death censored graft survival [345/364 (94.8%) vs. 15/20 (75%) vs. 24/25 (96%), *p* < 0.001] was significantly worse in patients after early CMV infection compared with either patients with no CMV infection or late CMV infection.

Significant differences in kidney function between patients with late CMV infection and other groups persisted at study completion. The median serum creatinine was 114 µmol/L in patients with no CMV, 123 µmol/L in those with early CMV, and 235 µmol/L in those with late CMV (*p* < 0.001).

## 4 Discussion

In this study, we compared 28 kidney transplant recipients who developed CMV infection ≥2 years post-transplant with 42 recipients who developed CMV infection within 2 years. Our findings suggest that the risk factors associated with late CMV infection are different from those associated with infection at earlier time points. Well-established risk factors for early CMV infection include donor-recipient serostatus (specifically D+/R−) (3), older donors (15), delayed graft function (16), a shorter course of prophylaxis (16), previous allograft rejection (17), and re-transplantation (18). Our findings suggest that renal dysfunction may be a risk factor for the onset of late CMV infection beyond 2 years post-transplant. The classically defined risk factors for CMV infection at earlier time points may be less significant in late infections. Differences in host immunity and acuity of viral exposure may underlie some of these differences. Early CMV infection arising after D+/R− transplantation may frequently represent primary infection in susceptible subjects after cessation of prophylactic therapy, whereas this dynamic is largely absent at later time points post-transplant.

In a prior retrospective cross-sectional study by Violet et al. (10), female sex, corticosteroid use, and a history of CMV drug-resistance mutation were identified as risk factors for asymptomatic CMV viraemia at two years post-transplant. Although baseline kidney function did not significantly differ between patients with and without CMV viraemia at the time of inclusion, those with viraemia experienced a greater decline in estimated glomerular filtration rate (eGFR) over the subsequent year. Multivariate analysis further revealed that CMV viraemia was independently associated with an increased risk of eGFR decline at one year. Notably, and consistent with our findings, D+/R− serostatus was not predictive of CMV viraemia at two years in their cohort.

The mechanisms by which kidney dysfunction may predispose to CMV infection remain speculative, but several possibilities bear consideration. Kidney impairment can result in reduced excretion of immunosuppressive agents and their metabolites, resulting in accumulation of active drug in affected patients. For example, severe renal impairment (creatinine clearance <25 ml/min) is associated with reduced excretion and enhanced enterohepatic recirculation of the main metabolite of the commonly used immunosuppressive medication mycophenolic acid-glucoronide (MPAG), which is re-activated and in turn leads to an increase in total and free MPA concentrations (19). In a study of 42 incident kidney transplant recipients predominantly treated with cyclosporin, higher MPA area under the curve (AUC) results were found to be associated with an increased risk of complications including that of opportunistic infection — one-third of which were CMV-related (20). Therefore, renal dysfunction at later post-transplant time points could potentially lead to elevated MPA concentrations, increasing the risk of infectious complications, including CMV infection. Furthermore, renal dysfunction may itself be independently associated with dysregulation of the innate and adaptive immune systems, which may lead to an increased propensity toward infection (21).

Establishing renal dysfunction as a defined risk factor for CMV infection would potentially allow for more accurate identification of at-risk patients and therefore targeted intervention to prevent and/or institute earlier treatment to improve outcomes and reduce complications (22). Patients with poor renal function may benefit from more frequent assessment of their immunosuppression exposures and potential reductions in doses. If available, measurements of MPA AUC can offer valuable information about current exposure for at-risk patients and allow for more tailored dose reductions. While patients treated with intermediate to high MPA AUC targets show significantly lower rejection rates compared to those with low targets (20), it may be appropriate to aim for lower targets in those patients identified to be at increased risk of viral infections such as CMV. In the absence of such measurements, at-risk patients may be considered for empirical reductions in total immunosuppression burden where felt to be appropriate.

The clinical significance of low level CMV positivity compared with higher thresholds is not clear. Our results suggest that CMV PCR <1,000 IU/ml may not necessarily foreshadow an increased risk of developing significant CMV infection <2 years post-transplant, as the rate of these episodes were similar in those who did not develop CMV infection compared to those with early infection. CMV “blips” were, however, significantly more common preceding CMV infection ≥2 years post-transplant. This may suggest that later infection can behave in a clinically distinct manner with a more subacute and gradual onset compared with that of earlier infection. The clinical implications of these findings require further study, and it is important to note that while CMV disease may be rarer in patients with lower viral loads, the impacts on graft and patient outcomes may still be significant.

The strengths of this study include the evaluation of patient data over an extended follow-up period and the inclusion of a significant number of kidney transplant recipients with late CMV infection—a population that remains poorly characterized in the existing literature. However, as a retrospective study based on existing clinical records, it was limited by incomplete data availability, and we were unable to determine certain clinical data such as the severity of CMV infection. Furthermore, the study required the use of arbitrary thresholds to define CMV infection (>1,000 IU/ml) and early/late time points (2 years), and it was challenged by the difficulty of comprehensively assessing dynamic parameters such as serum creatinine over time. Despite these limitations, the study provides valuable insight into the underexplored population of kidney transplant recipients with late CMV infection.

In conclusion, this single-center retrospective study demonstrates that late CMV infection (≥2 years post-transplant) is associated with risk factors distinct to those linked to earlier infection. Renal dysfunction may play an important role in the development of late infection and may overshadow the significance of donor-recipient serostatus. Further prospective studies are required to confirm whether renal dysfunction results in increased risk of late CMV infection, and to evaluate whether risk-based immunosuppression adjustments or targeted CMV surveillance could reduce complications and improve outcomes in long-term transplant recipients.

## Data Availability

The raw data supporting the conclusions of this article will be made available by the authors, without undue reservation.
