# Supplementary material for: Graft dysfunction is associated with late CMV infection after kidney transplantation
Source: Front Transplant. 2025 Nov 14;4:1647725. doi: 10.3389/frtra.2025.1647725 (PMC12660292; doi:10.3389/frtra.2025.1647725)

Supplementary Table 1. De-novo infection versus reactivation in early and late CMV infection

	**Early CMV**	**Late CMV**	***P***
**De-novo infection (R-)**	28/42 (66.7%)	6/28 (21.4%)	<0.001
**Reactivation (R+)**	14/42 (33.3%)	22/28 (78.6%)	

Supplementary Table 2. Comparison of the clinical characteristics between the patients with or without CMV infection; univariate analysis (patients with >1 positive CMV PCR).

	**No CMV**	**CMV infection**[1]		***P******
	(N=710)	**Early (<2 years)** (N=40)	**Late (>2 years)** (N=26)	
**Demographics**				
**Male**	441 (62.1)	22 (55)	15 (57.7)	0.612
**Age at Tx, years**[2]	50.7 (40.1, 60.3)	51.2 (39.9, 61.6)	44.2 (34.3, 57)	0.310
**Age at CMV infection, years**		51.7 (40.2, 62.2)	56.3 (40.4, 67.5)	0.310
**Graft number**				
1	587 (82.7)	32 (80)	24 (92.3)	0.390
>2	123 (17.3)	8 (20)	2 (7.7)	
**Transplant type**				
**Deceased Donor**	446 (62.9)	30 (75.0)	14 (50.8)	0.312
Donation after Brain Death (DBD)	322 (45.4)	23 (57.5)	12 (46.2)	
Donation after Circulatory Death (DCD)	124 (17.5)	7 (17.5)	2 (7.6)	
**Living donor**	264 (37.2)	10 (25)	12 (46.2)	
**Serostatus**				
D+/R-	105 (14.7)	28 (66.7)	2 (7.1)	<0.001
Others	595 (83.8)	14 (33.3)	23 (82.1)	
Unknown	10 (1.4)	0 (0)	3 (10.7)	
**Transplant characteristics**				
**Follow up time (days)**	1929	1486	4605	<0.001
**Time to CMV infection post-transplant (days)**		219 (129, 265)	3061 (2022, 5209)	<0.001
**HLA Mismatch**[3,4]	3.7 +/- 1.7	4.0 +/- 1.3	3.9 +/- 1.4	0.337
**Rejection**	42 (5.9)	7 (17.5)	3 (11.5)	0.010
**Lymphocyte count (10^9/L)**[5]	1.7 (1.2, 2.3)	1 (0.7, 1.8)	0.6 (0.4, 1)	0.001
**Tacrolimus (µg/L)**[6]	4.9 (3.9, 6.5)	6.5 (4.9, 7.9)	3.5 (2.6, 5.1)	<0.001
**BK virus infection**	144 (20.3)	12 (30.0)	5 (19.3)	0.631
**Time to BK virus infection post-transplant (days)**	90 (62, 185)	100 (62, 1253)	158 (78, 2670)	0.118
**Serum creatinine (µmol/L)**	113 (91, 144)	127.5 (97.5, 184.8)	234.5 (169.8, 362)	<0.001

Values are represented as number (%) unless otherwise stated

[1]Defined as blood CMV PCR ≥ 1000 IU/mL

[2]Median (IQR)

[3]Mean +/- SD

[4]HLA data missing in 3/710 CMV negative, 0/40 early CMV, 5/26 late CMV

[5]Tacrolimus data missing in 174/710 CMV negative, 3/40 early CMV, 10/26 late CMV

[6]Lymphocyte count data missing in 56/710 CMV negative, 20/40 early CMV, 12/26 late CMV

*Based on one-way ANOVA, chi-square test, Log-rank (Mantel-cox) test, two tailed student’s t-test

Supplementary Figure 1. Median time to onset of CMV infection.

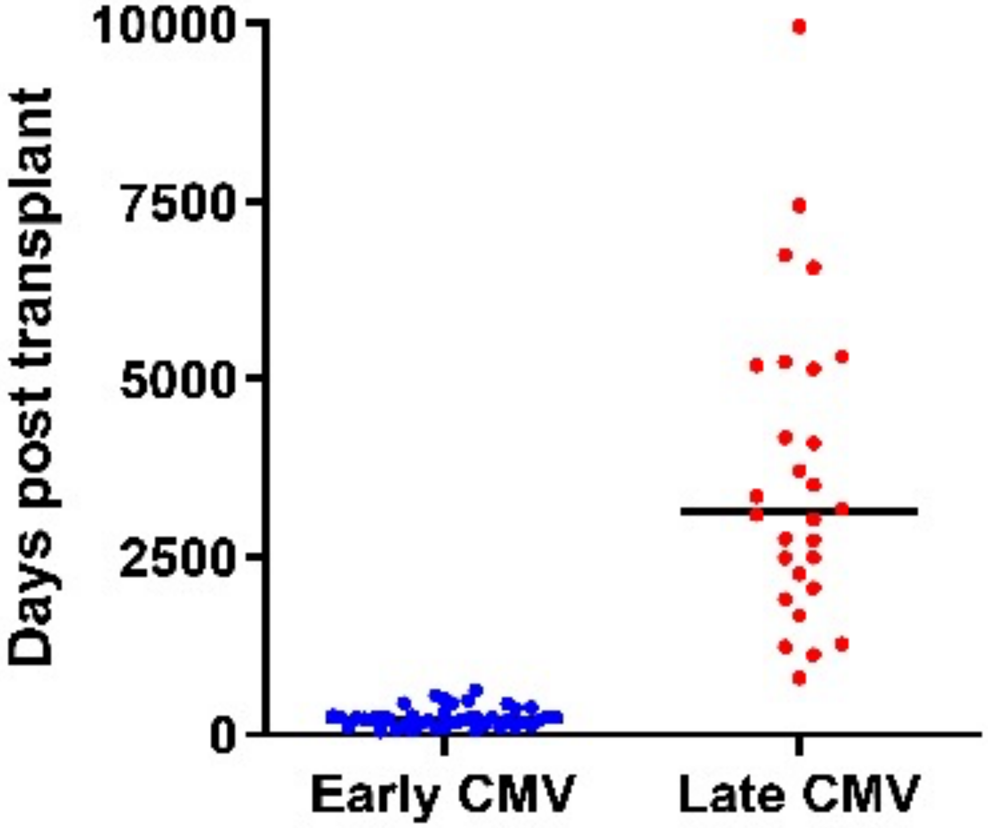

Supplement: Supplementary file 1 [file Datasheet1.pdf]
